# Chitosan/Silk Fibroin Materials for Biomedical Applications—A Review

**DOI:** 10.3390/polym14071343

**Published:** 2022-03-26

**Authors:** Anna Tuwalska, Sylwia Grabska-Zielińska, Alina Sionkowska

**Affiliations:** 1 Department of Biomaterials and Cosmetics Chemistry, Faculty of Chemistry, Nicolaus Copernicus University, Gagarin 7, 87-100 Toruń, Poland; planecka@doktorant.umk.pl; 2 Department of Physical Chemistry and Physicochemistry of Polymers, Faculty of Chemistry, Nicolaus Copernicus University, Gagarin 7, 87-100 Toruń, Poland; sylwiagrabska91@gmail.com

**Keywords:** chitosan, silk fibroin, blends, biomaterials

## Abstract

This review provides a report on recent advances in the field of chitosan (CTS) and silk fibroin (SF) biopolymer blends as new biomaterials. Chitosan and silk fibroin are widely used to obtain biomaterials. However, the materials based on the blends of these two biopolymers have not been summarized in a review paper yet. As these materials can attract both academic and industrial attention, we propose this review paper to showcase the latest achievements in this area. In this review, the latest literature regarding the preparation and properties of chitosan and silk fibroin and their blends has been reviewed.

## 1. Introduction

Biomaterials based on polymer blends have been widely studied because their potential in several applications has been increasing [1]. Blends can be prepared based on two or more synthetic polymers and also based on natural polymers. The blending process is usually considered when developing a new class of materials is expected. Especially, there is a need to fabricate new materials with better mechanical properties and biocompatibility for medical applications. In the biomedical field, the blending should lead to new materials with enhanced properties when compared with materials made of a single polymer. There are two main processes for blend preparation: blending in a molten state (so-called melt mixing) and/or dissolution in the same solvent. The next possibility of blend preparation is the joint action of high pressure and shear deformation of the solid component, but high pressure and temperature can destroy the structure of several biopolymers. For this reason, blends made from two or more different biopolymers are usually prepared by dissolution in the same solvent. However, sometimes another problem arises, namely, the low solubility of the components in the same solvent.

**Figure 1:**
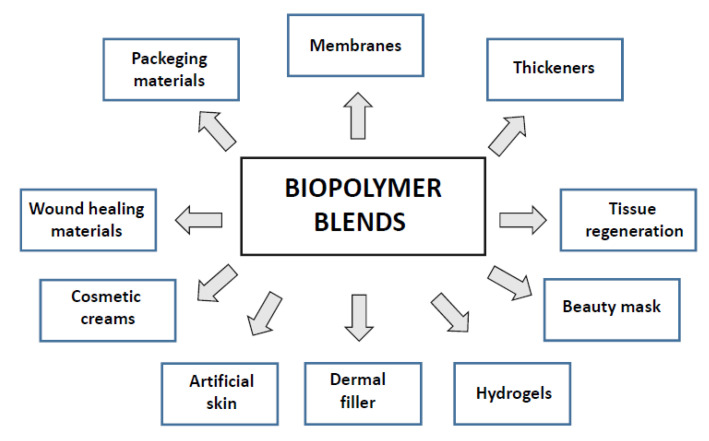
Potential materials based on biopolymer blends.

Within the last two decades, we can observe that new review papers regarding polymer blends can be found [2,3,4,5,6,7]. For example, Younas et al. published a comprehensive overview dealing with several natural and synthetic polymer modifications with cellulose as a reinforcer in respective polymer matrices [4]. Tabasum et al. published a review paper on blending corn starch with natural and synthetic polymers as well as inorganic nanoparticles [5]. In the following review written by Tabasum et al., the applications of blends and composites of pullulan with natural and synthetic polymers have been summarized [6]. A review on collagen blends with other biopolymers has been prepared by Sionkowska [2]. In particular, collagen and chitosan blends are widely studied [7,8,9,10,11,12]. Using biopolymer blends, several new materials can be proposed for both biomedical and cosmetic applications (Figure 1) [13,14,15,16,17,18,19,20,21,22,23,24,25,26,27,28,29,30].

It has been shown so far that several polysaccharides can be blended with different synthetic polymers and with other polysaccharides and proteins. In the scientific literature, one can find reports regarding the blends of carrageenan, mucilages, chitosan, hyaluronic acid, cellulose, sodium alginate, starch, and many others. In this review, the recent literature regarding the blend made from chitosan and silk fibroin has been presented. It is commonly known that chitosan is widely used for biopolymer and polymer blend preparation. There are many reports that show blends of chitosan with another biopolymer. The blends of chitosan and silk fibroin have also been widely studied. Both chitosan and silk fibroin can be used for biomaterial fabrication.

## 2. Chitosan

**Figure 2:**
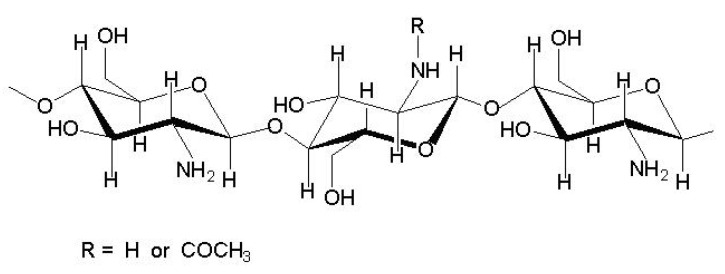
Chitosan structure.

Chitin is a polysaccharide comprised of 2-acetamido-2-deoxy-β-d-glucopyranose units. It is an insoluble biopolymer; however, from chitin, its soluble derivative, chitosan, can be obtained. In chitosan macromolecule, part of chains exists in the deacetylated form as 2-amino-2-deoxy-β-d-glucopyranose. CTS is defined when the degree of deacetylation is at least 50%. Chitosan is soluble in dilute acids, and after solvent evaporation, thin films can be formed. The structure of chitosan is shown in Figure 2. Chitosan is widely used in biomedical, pharmaceutical, and cosmetic applications because it is a biodegradable polymer. Moreover, CTS is biocompatible, possesses high charge density, and is non-toxic and mucoadhesive [31].

Chitosan is widely used in pharmaceutical and cosmetic applications due to its biocompatibility and biodegradability. However, CTS can also be used for medical products, wastewater treatment, biomembranes, and hydrogel development [31,32,33,34,35]. Many papers discuss the advances in the application of chitosan and chitosan derivatives to non-viral gene delivery and give an overview of transfection studies that have been performed recently using chitosans as transfection agents [36]. Chitosan can be modified to enhance the properties required in biomedical applications. After such modification, chitosan is a versatile biomaterial for cell therapy, tissue engineering, and gene therapy [37]. In biomedical applications, chitosan hydrogels and networks (for instance, interpenetrating polymer networks) formed by aggregation or complexation have been developed [38]. Materials prepared from chitosan can be used for drug delivery [39,40,41,42,43,44]. Cosmetic formulations often contain chitosan as a rheology modifier and film-forming agent. Several cosmetic products containing chitosan can be found on the market. Chitosan can be modified by covalent crosslinking, and it leads to the formation of hydrogels. Hydrogels with controlled network structures are widely used for the absorption of water and/or bioactive compounds. This can be further used for drug release by diffusion under pH-controlled conditions. The swelling sensitivity to pH changes depends on the kind of crosslinker, for example, ionically crosslinked chitosan hydrogels exhibit a higher swelling sensitivity to pH changes than covalently crosslinked chitosan hydrogels. This fact extends the potential application of ionically crosslinked chitosan [45]. Chitosan and its derivatives can be applied in various tissue engineering applications, namely, skin, bone, cartilage, liver, nerve, and blood vessel [46,47]. For cancer treatment, injectable chitosan hydrogels have been proposed [48,49,50].

Several review papers have been published within the last 5 years regarding chitosan materials [51,52,53,54,55,56,57,58,59,60,61,62,63,64,65,66,67,68,69,70]. According to the Web of Science^®^ Data Base, about 220 papers have been published in which the word «chitosan» and «review» appear in the title of the article. These data show that many scientists tried to review and summarize the complex knowledge about chitosan materials. A historical review of chitin and chitosan biopolymers was carried out by Crini [51]. It has been shown that chitin and chitosan research have a quite long history. A critical review of chitosan application in biomedical engineering has been done by Mohebbi et al. [52]. The emerging applications of chitosan in medicine, tissue engineering, drug delivery, gene therapy, cancer therapy, ophthalmology, dentistry, bio-imaging, bio-sensing, and diagnosis have been summarized. Chitosan and chitosan-based nanocomposites are widely used in drug delivery [53,54,55], in cancer therapy [56], and in bone tissue engineering [57]. Chitosan is also used in wound healing. The review by Liu et al. summarized the current effectiveness of chitosan in wound healing [58]. The applications of chitosan films and scaffolds in regenerative medicine have been reviewed by Rezaei et al. [59]. In the above-mentioned review, the most recent advances in chitosan films and scaffolds in terms of preparation techniques and modifying methods for improving their functional properties in tissue engineering, wound healing, and drug delivery have been discussed. The review of preparation and adsorption properties of chitosan and chitosan composites was published by Liu et al. [60]. In this paper, the research background of chitosan and modified CTS as an adsorbent was summarized, together with adsorption mechanism. The applications of chitosan and chitosan-based metallic nanoparticles in agrosciences have been reviewed by Chouhan et al. [61]. The mechanism for stimulation of plant immunity by metallic nanochitosan has been reviewed, and it was stated that chitosan metallic nanoparticles showed enhanced anti-pathogenic and plant-growth-promoting activity in comparison to bulk chitosan. A systematic review of glycosylated-chitosan derivatives has been done by Sacco et al. [62]. The applications of such derivatives in biomaterial design and drug delivery have been disclosed. The chemical and biological properties of chitosan provide a broad potential application in the textile industry. The properties of chitosan related to textile finishing have been presented by Zhou [63]. The following processes in which chitosan found applications have been summarized: dyeing, printing, and functional finishing. Chitosan nanoparticles play a relevant role in research on polymeric nanomaterials with biomedical and pharmaceutical applications. The preparation methods of chitosan nanoparticles have been reviewed by Caro-Leon et al. [64]. The review of current research on the mechanisms and factors affecting antibacterial activity, and application of chitosan nanoparticles, was carried out by Chandrasekaran et al. [65]. The overview regarding diverse materials used in developing innovative chitosan-based nanocomposite polymeric membranes for water purification was published by Spoiala et al. [66] and Vidal et al. [67]. The most important applications of chitosan-based nanocomposite polymeric membranes and their perspectives in water purification have been summarized there. Chitin and chitosan-based composites have also been used for energy and environmental applications [68] and in food applications [69]. Chitosan is widely studied over the world, and several sources for obtaining of this biopolymer are known. The review by Iber et al. summarized the various sources of chitin and chitosan [70]. This review shows the possible sources of chitin and chitosan for commercial applications. From the number of publications regarding chitosan, one can conclude that the interest in the research of this biopolymer and its modification is increasing and may lead to new interesting materials.

## 3. Silk Fibroin

**Figure 3:**
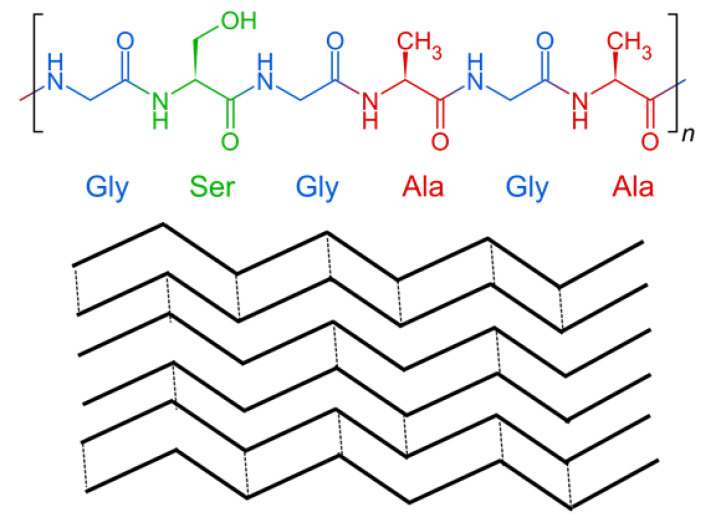
The primary and secondary structure of silk fibroin.

Silk fibroin is a biopolymer that is usually produced by the domesticated Bombyx mori silkworm. This biopolymer has been used as a suture material for many years. Silk fibroin is a fibrous protein produced not only by a variety of species, including silkworms, but also by spiders. Silk fibroin is a protein with primary and secondary structures. The primary structure is built of amino acids, and the molecule of silk fibroin consists of heavy chains and light chains. The secondary structure is usually β-pleated sheet and depends on the origin of silk fibroin. The structure of silk fibroin is shown in Figure 3.

**Figure 4:**
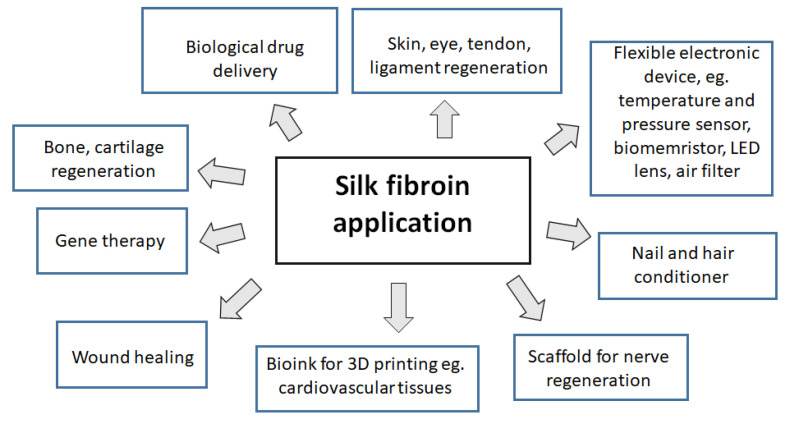
The most typical applications of silk fibroin.

Silk fibroin shows good biological compatibility and mechanical properties, so it is investigated for many biomedical applications [71,72,73,74,75]. It is widely studied by several research groups. Silk fibroin is a biocompatible material and, due to its slow degradation in vivo, can be used for the attachment and proliferation of many cells. From silk fibroin in the laboratory, we can obtain films and scaffolds. The above-mentioned forms can be used to improve regeneration of skin and nerve and in bone and cartilage tissue regeneration. Blending the natural silk fibroin with other polymers can lead to obtaining new materials. The recent applications of SF-based materials for small molecule drug delivery, biological drug delivery, gene therapy, wound healing, and bone regeneration have been reviewed by Nguyen et al. [76]. Silk-based materials can be fabricated by electrospinning process [77]. Several hydrogels for biomedical applications can be fabricated from silk fibroin [78,79,80]. Silk fibroin can be used as a functional biomaterial for drug and gene delivery [81,82,83,84,85]. The most typical applications of silk fibroin are shown in Figure 4.

Silk fibroin is also widely used for the fabrication of biopolymer blends for biomedical applications. The review paper about the possibility of blending and crosslinking of silk fibroin has been done by Grabska-Zielińska et al. [32]. Special attention has been paid to silk fibroin blends with collagen [86,87,88,89,90,91,92]. Using silk fibroin and collagen blend thin film and membrane [93], hydrogels [94], electrospun fibers [95,96], and hard connective tissue materials [97,98,99,100] can be prepared. Silk fibroin is also blended with other biopolymers. One of them is chitosan. The bends of chitosan and silk fibroin have been reviewed in the next chapter.

## 4. Blends of Chitosan and Silk Fibroin

### 4.1. Miscibility of Chitosan and Silk Fibroin Blends

A number of articles, both research and review, where authors describe mixtures of chitosan with silk fibroin, can be found in the literature [101,102,103,104,105,106,107,108,109,110,111,112,113,114]. The miscibility of this type materials was been studied by our research group some years ago [114]. Viscosity measurements of dilute polymer solution, using Ubbelohde capillary viscometer with two different criteria (Krigbaum and Wall [115], Garcia et al. [116]) to estimate the polymer–polymer miscibility in dilute solution, have been used in this research. The following weight compositions of the mixtures have been tested: 20CTS/80SF, 50CTS/50SF, and 80CTS/20SF, and it was concluded that chitosan and silk fibroin are miscible with the exception of the blend for high content of chitosan (w_Ch_ ≥ 0.8), according to the used criteria of polymer–polymer miscibility. Park et al. [110] came to the same conclusions during dynamic mechanical thermal analysis (DMTA) used for miscibility testing. They have been concluded that chitosan/silk fibroin mixtures were miscible in the range of 10–50 wt% chitosan content and that the blends exhibit considerable miscibility at the molecular level. Additionally, they stated that no trace of phase separation for chitosan/silk fibroin blends was observed by scanning electron microscopy (SEM) [110].

### 4.2. The Most Typical Forms of Chitosan/Silk Fibroin Mixtures

**Figure 5:**
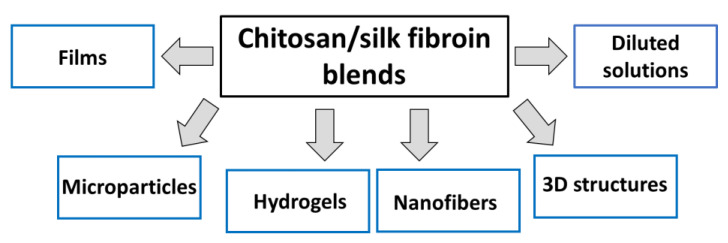
The most typical forms of chitosan/silk fibroin mixtures.

Apart from the tests of mixtures of chitosan with silk fibroin in the form of diluted solutions, nanofibers [112,117,118,119], three-dimensional structures [101,108,109,120,121,122,123,124,125,126,127,128,129], films [103,105,110,114,130,131,132,133], microparticles [134,135,136,137], and hydrogels [138,139,140] were also obtained and characterized (Figure 5).

### 4.3. Chitosan/Silk Fibroin Composites Nanofibres

Cai et al. [117] and Lai et al. [112] have been described chitosan/silk fibroin composites nanofibers for wound-dressing applications and bone tissue development obtained by electrospinning. The first group have noticed that the CTS/SF nanofibrous membranes could be developed and used as a product in wound healing treatment because they have been characterized by good physicochemical properties from the tissue engineering point of view. The addition of silk fibroin to chitosan enhanced the mechanical resistance of composite nanofibrous membranes and caused an increase in the diameter of nanofibers. On the other hand, with the increasing ratio of chitosan in the material, the antibacterial activity increased in relation to Gram-negative bacteria *E. coli*. Based on the biological tests, it was found that chitosan/silk fibroin materials promote cell attachment and proliferation [117]. In turn, the results of biological tests obtained in the second research group [112] suggest that chitosan and silk fibroin were excellent candidates for proliferation and osteogenic differentiation of hMSCs (human bone marrow mesenchymal stem cells), respectively. He et al. [118] performed a biological experiment for chitosan/silk fibroin nanofibers. They reported that the addition of chitosan to silk fibroin promoted cell (HRMCs-human renal mesangial cells) attachment and a spreading on silk fibroin nanofibers significantly. Chen et al. [119] used human fetal osteoblastic cells (hFOB) for cell culture analysis, and they reported that each component of materials, i.e., chitosan and silk fibroin, has a distinct effect on cell behavior. Chitosan enhances osteogenic differentiation and silk fibroin promotes cell proliferation [119].

### 4.4. Chitosan/Silk Fibroin Three-Dimensional Scaffolds

Three-dimensional scaffolds are a form of biomaterials, widely described in the literature and used in tissue engineering. In our previous article from a few years ago, we described chitosan/silk fibroin scaffolds in 80/20, 50/50, and 20/80 (*w/w*) ratio [109]. FTIR spectroscopy analysis, swelling properties, porosity measurements, mechanical properties, thermogravimetric analysis, and in vitro tests were performed. Additionally, SEM observations have been evaluated [109]. The scaffolds have been cross-linked by EDC/NHS mixture. We reported that thermal stability of two-component scaffolds was better than the stability of pure components. The pore size in CTS/SF materials was from 20 to 150 µm, and the mechanical integrity of scaffolds was sufficient to resist handling during implantation and in vivo loading in dry and wet state (PBS media). The obtained materials were biocompatible with the fibroblast 3T3 [109]. In a different manner, Bhardwaj and Kundu [120] have been obtained SF/CTS blend ratios (volumetrically) 2:1, 1:1, 1:2, and 1:3 scaffolds. SEM observations led to the conclusion that interconnected porous structures with 100 to 155 µm size pores were obtained. All tested scaffolds showed porosity ranging between 80 and 95%. FTIR spectroscopy showed new bands that were not present in either chitosan or silk fibroin, which was indicative of interactions between them [120]. Additionally, scaffolds showed higher compressive strength and modulus than pure components, antibacterial effect, and suitable degradation properties. Furthermore, the in vitro cytocompatibility tests revealed that CTS/SF-based materials facilitate the growth, attachment, and adhesion of feline fibroblast cells [120]. She et al. [121] chose HepG2 cells (human hepatoma cell line) for in vitro tests, and they also reported that CTS/SF scaffolds could promote proliferation significantly. They obtained the porosity, morphology, and mechanical strength tests, consistent with the above-cited articles [121]. Vishwanath et al. [122] found that the 80SF/20CTS scaffold is the best for use in cartilage tissue engineering because it was characterized by desired morphological pore size (range 71–210 µm), porosity (82.2 ± 1.3%), compressive strength (190 ± 0.2 kPa), degradation properties, and biocompatibility with MSC (mesenchymal stem cells) derived from umbilical cord blood. According to Gobin et al. [123], with increasing content of silk fibroin in material, it was observed that the ultimate tensile strength and elastic modulus increase significantly and increase the content of the chitosan, resulting in an increased water capacity of SF/CTS scaffolds. Furthermore, the degradation process slightly affects SF/CTS materials’ swelling properties, the degradation process runs much faster in the first 2 weeks, and the SF/CTS scaffolds can retain their porous structure till 6 weeks of degradation [124]. It can be found in the literature that scaffolds made of silk fibroin and chitosan can be potentially used in bone [101,108,109], cartilage [125,126], wound [127,128], and tissue engineering, and that cytocompatibility of the CTS/SF scaffolds was referenced with the following cells: MG-63 [101], NCTC clone 929 cells [127], hMSCs (human mesenchymal stem cell) [126], and rabbit corneal keratocytes [129].

### 4.5. Chitosan/Silk Fibroin Films

Films are a very popular form of chitosan and silk fibroin materials, in addition to three-dimensional structures. So far, various physico-chemical properties of chitosan and silk fibroin films have been investigated, including FTIR spectroscopy structural measurements, DSC measurements, thermogravimetric analysis, surface and cross-section morphology by SEM, XRD tests, mechanical properties, contact angle measurements, percent moisture uptake, AFM topographic imaging, water vapor permeability, swelling property, or enzymatic degradation [105,110,111,114,130,131,132,133]. Additionally, biological tests of CTS/SF-based films have been evaluated. It was found that blending of chitosan with silk fibroin can enhance the thermal decomposition stability of material [130], and when silk fibroin proportion in the material increases, the temperature at which degradation is initiated also increases [131]. According to our previous paper about physical properties of chitosan and silk fibroin mixtures [114], ultimate tensile strength, Young modulus, and tensile strain at break depended on the weight fraction of chitosan and silk fibroin and were changed irregularly. When the w_CTS_ ≤ 0.5 (w_CTS_-weight ratio), the value of tensile strength was about 15% less than that evaluated for pure chitosan film [114]. Li et al. [132] suggested that the CTS/SF thin films could be promising materials for tissue engineering of bone, adipose, cartilage, and skin because they were remarkably biocompatible and were characterized by appropriate hydrophilicity and mechanically resistance. In this study, rat bone marrow-derived mesenchymal stem cells have been used for biological tests [132]. They have been reported that thin films based on CTS/SF mixtures provided a comparable environment for the growth and proliferation of cells and promoted their osteogenic and adipogenic differentiation [132]. Regarding the coefficient of water vapor permeability of the CTS/SF films, Kweon et al. [111] reported a linear increase with the chitosan content. They obtained values of 1000–2000 g∙m^−2^∙day^−1^, which was comparable with values of commercial wound dressings [111]. The chitosan and silk fibroin content in the blend films had an influence on density and swelling degree [111], and all tested films showed no cytotoxicity with the HDF (human dermal fibroblasts) cells. Based on these results, CTS/SF thin film can be used as metal or ceramic implant surface coating for bone injury repair and as tissue engineering scaffold for cornea, adipose, skin, and other soft tissue injury repair [110,111,114,130,131,132,133].

### 4.6. Chitosan/Silk Fibroin Microparticles

There are also several reports in the literature where scientists prepared microparticles based on the mixtures of chitosan and silk fibroin [134,135,136,137]. They have been prepared, among others, by the W/O (water in oil) emulsification-diffusion method [134]. The CTS/SF microparticles, in contrast to SF microparticles, were spherical with a rough surface texture, and their average size was in the range of 73 to 80 µm. The CTS/SF blend ratio had an influence on dissolution, porosity, density, and structure of microparticles [134]. Liu et al. [135] obtained CTS/SF microspheres with the precipitation/coacervation method for use as a release delivery system for DNA vaccine. Moreover, CTS/SF microspheres cross-linked with genipin might function as effective drug carriers [137].

### 4.7. Chitosan/Silk Fibroin Hydrogels

To process chitosan/silk fibroin hydrogels for biomedical applications, ionic liquids have been used [138]. Biological tests in this study demonstrated that CTS/SF hydrogels supported the growth and adhesion of primary human dermal fibroblasts [138]. CTS/SF hydrogels cross-linked with EDC/NHS, prepared as a prospective material for sustained drug release, had porous structure, stable mechanical strength, stimuli responsive swelling performance, and drug release behaviors [139]. However, CTS/SF hydrogel constitutes an excellent candidate for wound dressings because it presents higher swelling and in vitro degradation rates under acidic conditions than in a neutral environment, exhibits good bacteriostatic and hemostatic properties, and is non-toxic towards human skin fibroblasts [140].

### 4.8. Cross-Linking of Chitosan/Silk Fibroin Materials

The above-mentioned results concerned mostly unmodified chitosan- and silk-fibroin-based materials. It is worth mentioning that cross-linking can be used to improve physico-chemical properties of such materials. Chemical, physical, and enzymatic methods are using to biopolymeric materials modifications [32]. Few cross-linking agents have been used to modify physico-chemical properties of CTS/SF materials. Genipin [137,141], glutaraldehyde [104,142,143] and the mixture of EDC (*N*-(3-dimethylaminopropyl)-*N*′-ethylcarbodiimide hydrochloride) and NHS (N-hydroxysuccinimide) [109,139] are the typical agents to cross-link CTS/SF-based mixtures. Materials cross-linked with genipin have been characterized as stable and ordered structures with different pore sizes and distinct morphologies, related to the chitosan ratio [141]. The cross-linking with genipin promoted the formation of stable structures that favored adhesion, proliferation, and matrix production of mouse fibroblast-like cells (L929 cells) [141]. Zeng et al. [137] investigated chitosan/silk fibroin microspheres cross-linked with genipin. They have found that these microspheres might function as effective drug carriers because they have been characterized by regular surface morphology, an appropriate particle size, high encapsulation efficiency, optimal physical and chemical properties, and prolongated release [137]. Glutaraldehyde has been used as cross-linker of chitosan/silk fibroin blend films for drug delivery system [104]. Rujiravanit et al. [104] found that the maximum amounts of drug released were obtained from the film with 80% of chitosan and 20% of silk fibroin. The mixture of EDC and NHS has been used to cross-link chitosan/silk fibroin composite sponges for tissue engineering [109] and environmentally sensitive hydrogels as drug carriers for sustained drug release [139]. Sionkowska and Płanecka [109] obtained three-dimensional structures with interconnected pores, proper swelling ratio, good mechanical properties, and better thermal stability than non-crosslinked materials. Additionally, studied materials have been biocompatibile with 3T3 fibroblasts [109]. On the other hand, Xu et al. [139] obtained hydrogels with microporous and mesporous structures in the internal structure. Prepared materials showed good mechanical properties, stimuli-responsive swelling performance, and drug-release behaviors [139].

**Figure 6:**
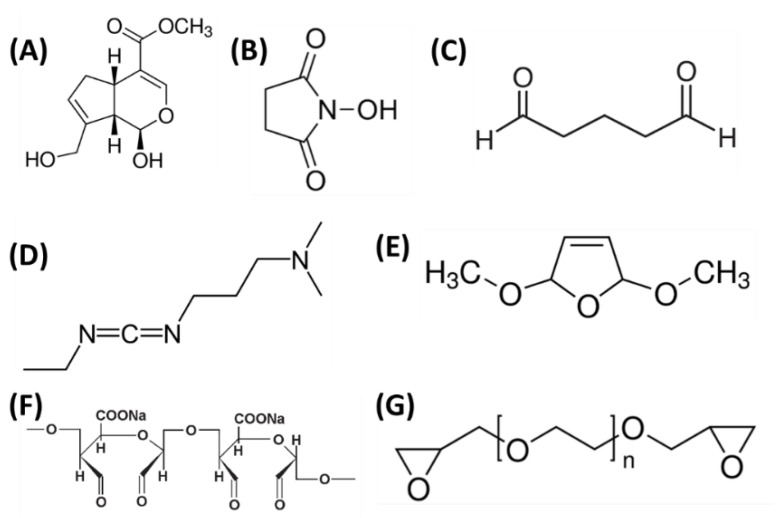
Agents used to chitosan/silk fibroin mixtures cross-linking: (**A**) genipin; (**B**) N-hydroxysuccinimide (NHS); (**C**) glutaraldehyde; (**D**) 1-Ethyl-3-(3-dimethylaminopropyl) carbodiimide hydrochloride (EDC); (**E**) 2,5-Dihydro-2,5-dimethoxyfuran (DMDF); (**F**) alginate dialdehyde (ADA); and (**G**) polyethylene glycol diglycidyl ether (PEGDE).

However, there are also reports of using DMDF (2,5-Dihydro-2,5-dimethoxyfuran) [144], ADA (alginate dialdehyde) [145], and PEGDE (polyethylene glycol diglycidyl ether) [146] as cross-linkers. The structural formulas of the cross-linking agents used for chitosan/silk fibroin mixtures are shown in Figure 6.

### 4.9. Combination of Silk Fibroin with Chitosan Derivatives

**Figure 7:**
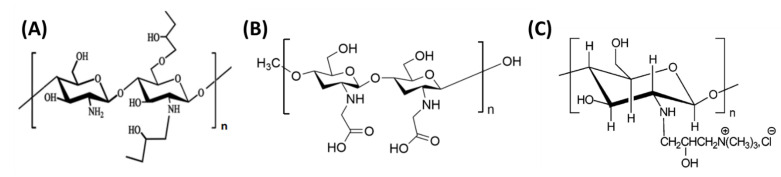
The structural formulas of chitosan derivatives: (**A**) hydroxybutyl chitosan; (**B**) carboxymethyl chitosan; and (**C**) quaternary ammonium chitosan.

This chapter was devoted to mixtures of chitosan and silk fibroin. However, it is also worth mentioning that there are reports in the literature where the constituents of the two-component mixture, in addition to fibroin, are chitosan derivatives (Figure 7). Combinations of silk fibroin with the following chitosan derivatives are known: hydroxybutyl chitosan [147], carboxymethyl chitosan [148,149,150,151,152], and quaternary ammonium chitosan [153].

## 5. Composites Based on Chitosan, Silk Fibroin, and Inorganic Particles

### 5.1. Composite Fabrication

**Figure 8:**
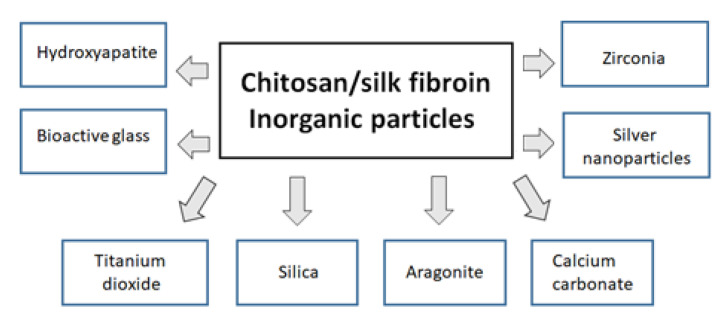
The examples of inorganic particles to be used in preparation of composites based on a blend of chitosan and silk fibroin.

New materials for biomedical applications can contain inorganic particles and/or nanoparticles. The inorganic compounds can be added into biopolymers and biopolymer blends but can also be formed by the precipitation of the inorganic phase in the biopolymer matrix to form the hard phase. During the precipitation process, the hard phase is created, which can be strengthened by the minerals. The minerals can be formed in different sizes, shapes, and distributions of individual crystals. The most important inorganic compounds that can form mineral phases in composites for biomedical application are hydroxyapatite, silica, and aragonite [154]. In the laboratory, one can try to fabricate a composite based on the blends of two or even more biopolymers containing inorganic particles. The self-organization of natural hard tissue outside the living body is difficult to mimic in laboratory conditions. Nevertheless, the development of new materials based on the blends of two or more polymers and inorganic nanoparticles has been started by several research groups. The incorporation of nanoparticles into the mixtures of two polymers or biopolymers leads to new materials that can be used as an implant of both hard and soft tissues, but they have to meet several requirements. The examples of inorganic particles to be used in composite preparation are shown in Figure 8.

The intercalation of inorganic nanoparticles in a polymeric matrix may lead to several nanostructured materials. However, it should be emphasized that in several cases, the fabrication of such materials is not easy because of the insolubility of natural polymers in water and common solvents [155,156]. When we consider chitosan and silk fibroin blends, we can say that it is easy to get the solution of both of these biopolymers in acidic pH. Such solutions, after characterization, can be used to design a polymeric matrix for the incorporation of an inorganic phase. For example, chitosan/silk fibroin blend films containing nanoparticles of methoxy poly(ethylene glycol)-β poly(d,l-lactide) were prepared by film casting of nanoparticle suspension-CTS/SF blend solution [105]. The nanoparticles with spherical shape (less than one μm) were observed from SEM micrographs. It was shown that intermolecular bonds between nanoparticles and CTS/SF film matrices appear in the material. The nanocomposite blend films containing nanoparticles exhibited an increase of film strength and decrease of film flexibility and water wettability.

### 5.2. Chitosan/Silk Fibroin and Hydroxyapatite

Chitosan/silk fibroin blend has been used by several researchers for the fabrication 3D composites containing hydroxyapatite (Hap) [157,158,159,160,161,162,163,164,165,166,167,168,169,170,171,172,173,174,175,176]. A new material based on hydroxyapatite/chitosan-silk fibroin (Hap/CTS-SF) in the form of the composite was prepared for bone repair and replacement by a co-precipitation method by Wang et al. [157]. The results of the above report revealed that the inorganic phase in the composite was carbonate-substituted Hap, which showed low crystallinity. The shape of Hap crystallites was needle-like, 20–50 nm in length, and around 10 nm in width. The compressive strength of the composite was higher than that for the precipitated Hap, without any organic source involved. This fact was closely related to the perfect incorporation of chitosan and SF macromolecules into the composite. It has been stated that the chemical interactions that occur between the mineral phase and the organic matrix may lead to improvement of the interfacial bonding, which can further lead to the enhanced mechanical properties of the composite.

The porous scaffolds of silk fibroin-chitosan/nano-hydroxyapatite (SF-CTS/n-Hap) were fabricated through the freeze-drying technique by Wen et al. [158]. It has been shown that the porous structure was formed and that chemical binding is formed between Hap and biopolymers. The obtained composite had high porosity of 78–91%, compressive strength of 0.26–1.96 MPa, and good bioactivity [158]. It has been suggested that composite scaffolds can be used for bone tissue engineering. Other research by Wang et al. [159] shows that a new composite can be formed by the introduction of silk fibers into hydroxyapatite (Hap)/chitosan matrix. In such a way, a scaffold material for bone tissue engineering with adequate initial strength and improved cellular affinity was fabricated using a combination of in situ synthesis and freeze-drying technique. The obtained composite had a pore size of 100∼250 μm and a porosity of 75∼90%. The maximum compressive strength was 5.7 MPa, and it was bigger than that for Hap/CTS matrix (4.6 MPa). The cell culture experiments showed good bioactivity and cellular compatibility of the composite material, so the potential for bone tissue engineering is high. The research by He et al. [160] shows that tussah silk fibroin (TSF) can also be used for composite fabrication. For biomimetic synthesis as the precursors of inorganic phase, Ca(NO_3_)_2_ and Na_3_PO_4_ were used together with TSF and CTS. As a result, new nanocomposite particles of hydroxyapatite-tussah silk fibroin were fabricated. In the nanocomposite particles, the carbonate-substituted Hap with low crystallinity was obtained. It has been shown that TSF and CTS induced preferential alignment of Hap crystallites along the direction of c-axis, and the induction effect of TSF was more evident than of CTS. It was found that Hap-TSF and Hap-CTS nanocomposite particles have a “needle-like” shape. The typical size was 100–200 nm in length and about 20 nm in width for Hap-TSF, whereas for Hap-CTS the size was 115–250 nm in length and about 25 nm in width. Using the fabricated nanocomposite particles, the bone-like nanocomposites of Hap-TSF/CTS and Hap-CTS/TSF with the same compositions were prepared by isostatic pressing using CTS and TSF concentrated solutions as adhesive composition, respectively. It was found that the compressive strength, compressive modulus, and bending strength of Hap-TSF/CTS composite were significantly higher than that of Hap-CTS/TSF composite.

The porous scaffold made from chitosan, silk fibroin, and nanohydroxyapatite particles (nHap) was prepared via salt fractionation method combined with lyophilisation by Qi et al. [161]. In this study, a porogen (salt) was used to fabricate the porous structure. The pore size was controlled by the size of the porogen. The scaffolds fabricated in this study had pore sizes of 100–300 μm and a porosity of 90.5–96.1%. For fabricated scaffolds, mechanical properties were measured. It was found that the tri-component scaffold has better properties than the bi-component scaffolds. Moreover, a biocompatibility was studied using the osteoblast-like MG-63 cells. The results showed that all the scaffolds are suitable for cell attachment and proliferation. The properties of 3D scaffolds can be modified by a crosslinking process. The three-dimensional silk fibroin/chitosan/nanohydroxyapatite scaffolds were prepared by repeated freeze-drying and chemical crosslinking technology by Ye et al. [162]. It was found that with the increased content of nanohydroxyapatite, the porosity, water absorption rate, and average pore size of the scaffolds decreased, while the hot-water loss rate and compressive strength increased. The scaffold prepared with the ratio 1:1:1 was better for bone tissue engineering than other ratios.

An interesting procedure for composite fabrication was published by Zhou et al. [163]. In the above-mentioned procedure, chitosan/nanohydroxyapatite composites were prepared in the first step. In the second step, the composites were used for fabrication of four-layer porous scaffold. Such a scaffold was prepared for potential biomedical application as articular cartilage repair. Chitosan/Hap composite was used for building the bottom layer, whereas the other three layers of the scaffold were fabricated using chitosan/SF composites. In the above-mentioned composites, the content of the chitosan and SF was altered in a mutually reversed trend. Moreover, the crosslinking of scaffolds using tripolyphosphate was performed. The porosity and mechanical and biological properties of the scaffolds showed that chitosan/SF/Hap scaffolds have promising potential for articular cartilage repair.

The nanofibrous membrane scaffold (NMS) based on nanohydroxyapatite (nHap) within a chitosan/silk fibroin has been proposed by Lai et al. [164]. In this study, two kinds of composites were prepared: a pristine CTS/SF NMS composite, and composite CTS/SF/nHap/NMS containing intrafibrillar nHap by in situ blending of 10% or 30% nHap before the electrospinning step. Moreover, a composite CTS/SF/nHap NMS containing extrafibrillar nHap by depositing 30% nHap through alternative soaking surface mineralization was prepared. It has been shown that the alternative soaking surface mineralization method drastically influenced the mechanical properties of the NMS, with 88% and 94% drop in Young’s modulus and ultimate maximum stress. The biological properties of the scaffolds showed the potential for bone regeneration and possible application for bone tissue engineering. Not only silk fibroin but also silk sericin was studied for composite preparation. Chitosan-silk sericin/hydroxyapatite nanocomposites by in situ precipitation were developed by Chen et al. [165]. In fabrication of composite, the mixing ratio of hydroxyapatite, silk fibroin, and chitosan is crucial to get appropriate properties for biomedical applications [166,167,168].

The latest scientific reports show that there are still huge interests in chitosan, silk fibroin, and hydroxyapatite composites. Xiao et al. [169] proposed new scaffolds with gradient pore sizes fabricated with silk fibroin, chitosan, and nano-hydroxyapatite. The series of porous nanohydroxyapatite/silk fibroin/chitosan scaffolds has also been fabricated in our research group using the freeze-drying method [170].

The effect of different treatment methods of silk (degummed, un-degummed, or dissolved SF) and different contents of SF on the properties of the CTS/n-Hap composite membrane was investigated by Tang et al. [171]. The research results showed that the degummed SF/CTS/n-Hap composite in the form of a membrane with a weight ratio of 2:6:2 had the highest mechanical strength. In the membrane, SF supported the structure as a skeleton frame. However, the un-degummed SF and dissolved SF lead to a weaker reinforce effect. This was due to the poor interface or poor interaction between SF and CTS. The dissolved SF/CTS/n-Hap composite membrane showed the fastest degradation rate. It has been summarized that all three SF may lead to the improvement of the cell biocompatibility of the CTS/n-HA composite membrane.

A key to bone formation and repair is bone morphogenetic protein-2 (BMP-2). The possibility of loading the silk fibroin/chitosan/nanohydroxyapatite scaffold with loading BMP-2 has been studied by Huang et al. [172]. The porous scaffolds were prepared with the appropriate release rate of BMP-2. Chitosan/silk fibroin/hydroxyapatite composites can be fabricated by in situ precipitation method. Ran et al. [173] synthetized chitosan-tussah silk fibroin/hydroxyapatite composites with the elastic modulus ranging from ∼250 to ∼400 MPa and fractured strength ranging from ∼45 to ∼100 MPa. The above-mentioned composite can be used as a scaffold platform for cell culture and implantation of bone reconstruction. Wu et al. [174] prepared silk fibroin and hydroxyapatite material incorporated into chitosan/glycerophosphate (GP) system to prepare new types of injectable hydrogels.

Although there is much research around the world regarding chitosan, silk fibroin, and hydroxyapatite composites, the application of such materials is in a very early stage. It is commonly known that all new biomaterials require proof that they are safe and effective before they can be approved for marketing. For some research cited previously, several in vitro tests have been provided. The research by Ye et al. [175] has been done to investigate the capacity and mechanism of silk fibroin, chitosan, and nanohydroxyapatite composite scaffold in repairing bone defects. In this research, 45 New Zealand white rabbits were used to model defect in the right radial bone. At 3–4 months after modelling, the X-ray scan and histopathological observation indicated that the normal bone tissues completely replaced the SF/CTS/nHap scaffold with an unobstructed marrow cavity. Therefore, it was concluded that the SF/CTS/nHap composite scaffold might be a promising material for bone tissue engineering.

### 5.3. Chitosan/Silk Fibroin, Metal/Metal Oxides, and Bioactive Glass Composites

Apart from the research regarding chitosan, silk fibroin, and hydroxyapatite composites, other inorganic compounds have been used to prepare composite. Wu et al. [176] proposed the use of calcium carbonate (CaCO_3_). The crystallization of the above-mentioned compound was investigated using a mineralization system. The system was composed of a chitosan membrane and regenerated silk fibroin. The results showed that on the chitosan membrane, the vaterite disks were mainly formed. When the pH value or temperature of the solution had been changed, then the aragonite disks were prepared. These disks consisted of nanoparticles about 20 nm in size. The crystallization of CaCO_3_ in the vicinity of the chitosan membrane was much more affected by the environment of crystallization, compared to that in bulk solution. Next, composite materials based on silk fibroin and chitosan TiO_2_ were fabricated [177]. Bombyx mori silk was modified with the nano-TiO_2_ and chitosan dispersion system by the crosslinking reactions. For the crosslinking, citric acid and maleic anhydride were used. The average size of the nano-TiO_2_ particles in the aqueous dispersion system was 36.7 nm. The shapes of nano-TiO_2_ particles were spherical. Moreover, nanoparticles were homogeneously dispersed in the dispersion system. The surface roughness of silk fiber treated with the nanoTiO_2_ and chitosan dispersion system was bigger than that of the untreated one [177]. It was found that the mechanical properties, including breaking strength, breaking elongation, initial modulus, rupture work, and elastic recovery property of the silk fiber in the presence of nano-TiO_2_, were significantly enhanced.

Silver nanoparticles are often applied as coatings on biomaterials and can be added into the bulk of biomaterial because they show antimicrobial properties. Karthikeyan et al. [178] reported that silk fibers can be coated with chitosan impregnated with silver nanoparticles, and in such a way antimicrobial activity and improved thermal stability can be reached. Such a composite can be considered as wound dressing and tendon reconstruction material in future. Chitosan-silk fibroin composite scaffolds containing silver nanoparticles (AgNPs) were fabricated for tissue engineering dressings by Liu et al. [179]. The materials fabricated in such a way showed good antimicrobial properties and can thus be considered for application in wound dressing. Next, composite materials based on silk fibroin and chitosan zirconia ZrO_2_ were incorporated. The study by Teimouri et al. [180] showed that silk fibroin and chitosan and zirconia ZrO_2_ can be combined using the freeze-drying technique to fabricate a bio-composite porous scaffold with good cytocompatibility and mechanical properties. The research by Li et al. [181] showed that new composite can be fabricated based on bioactive glass, silk fibroin, and chitosan. A biocompatible bioactive glass-based scaffold for bone regeneration applications with long-lasting antibacterial activity was fabricated by Zhou et al. [182].

In tissue engineering magnetic nanoparticles have been of great interest during the last few years. It is because of their controlled responsive characteristics in specific external magnetic fields. Silk fibroin/chitosan-based magnetic scaffolds were prepared using different amounts of magnetite nanoparticles by freeze-casting method by Aliramaji et al. [183]. It has been shown that the lamellar structured scaffolds with magnetic nanoparticles in the walls had good biological characteristics. The magnetic responsivity was added to the scaffolds and may lead to new, interesting materials.

Composite materials can be also fabricated by electrospinning and electrostatic layer-by-layer self-assembly. In the research by Chen et al., silk fibroin, chitosan, and rectorite composite mats were prepared by electrospinning technique [184]. Electrospun silk fibroin was selected as the substrate, and chitosan and rectorite were assembled on the surface of the substrate as positively and negatively charged layers via electrostatic layer-by-layer method. The cell-culture experiments demonstrated that the mats maintained superior biocompatibility after the introduction of chitosan and rectorite.

A chitosan/silk fibroin blend was used for incorporation of zinc oxide nanoparticles by Salama [185]. Because of the antibacterial activity of zinc oxide, the composite can be considered as material for wound dressing.

To produce new composites, the strontium-substituted hydroxyapatite was used together with silk fibroin, carboxymethyl chitosan, and cellulose nanocrystals [186]. The fabricated scaffolds had a porous sponge-like structure, with porosities over 80%. A significant increase in compressive strength of scaffolds containing strontium substituted hydroxyapatite was observed in comparison to silk fibroin and carboxymethyl chitosan scaffold.

Another example of new composite fabrication is preparation of Mg(OH)_2_ nanoparticles and incorporation of them into biopolymer matrix. A novel nanobiocomposite scaffold based on modifying synthesized cross-linked terephthaloyl thiourea-chitosan hydrogel substrate using the extracted silk fibroin biopolymer and prepared Mg(OH)_2_ nanoparticles was proposed by Eivazzadeh-Keihan et al. [187]. The biological capacity of this nanobiocomposite scaffold was evaluated by cell viability method, red blood cells hemolytic, and anti-biofilm assays. It was found that the scaffolds had good biocompatibility and mechanical properties.

Silica nanoparticles were also used as inorganic component in the composite based on chitosan and silk fibroin. Amino-functionalized mesoporous silica nanoparticles with radially porous architecture were synthesized by Yu et al. [188] and further used together with silk fibroin and chitosan to produce a type of covalently crosslinked composite hydrogel using genipin as a crosslinker. The obtained gels showed the ability to release bioactive Si ions suited to an effective dose range in approximately linear manners for a few weeks. The obtained composite gels have promising potential in bone repair and regeneration due to their osteogenic capacity. Inorganic phase in composites can be formed by nanoclays. In the work by Chen et al. [189], the effects of two nanoclays, montmorillonite and sepiolite, on the properties of chitosan and chitosan/silk peptide films were compared. The proposed composite was considered as material for wound dressing. Medical dressings blended with silk fibroin, chitosan, and halloysite nanotubes (HNTs) were successfully prepared by electrospinning by Ren et al. [190]. The broad-spectrum antibacterial drug was initially loaded into HNTs. The addition of HNTs had a significant effect on the micro morphology of the nanofibers and the thermal stability and tensile properties of electrospun materials. Such micro-nanofiber medical dressing prepared by electrospinning showed stable mechanical strength, rapid hemostasis, and antibacterial activity and thus has potential for biomedical application.

## 6. A Critical Comparison of the Existing Knowledge in the Field of Chitosan/Silk Fibroin Blends

According to the Scopus^®^ Data Base, about 730 papers have been published in which the word «chitosan» and «silk fibroin» appears in a title, keyword, or abstract (only in an article title—about 300). These data show that many research groups have been working with chitosan and silk-fibroin-based materials. However, only about 33 papers discuss blends of chitosan and silk fibroin when we use the words «chitosan», «silk fibroin», and «blend» in order to find results. The results concern only the combination of the keywords in titles. When the search takes abstracts into account, there are more results, about 110 documents. These data were collected in February 2022. Probably, there are also reports not provided by Scopus^®^, so they are not easily accessible.

Although there is much research regarding chitosan/silk fibroin blends, the application of such blends and commercialization of the new materials based on such a blend is rather at a very early stage.

When comparing the results and existing knowledge about materials based on chitosan and silk fibroin blends, it was noticed that new materials had been fabricated in the form of membranes and 3D forms. A protocol for blends preparations can differ from one laboratory to another, so sometimes it is not easy to compare the results. Moreover, in several papers the molar masses and deacetylation degree of chitosan used in the research have not been discussed. Usually, low-molecular-weight chitosan was used because it is easier to dissolve and use to fabricate biomaterials. Nevertheless, most of published papers show that materials fabricated from chitosan/silk fibroin blend are biocompatible and possess mechanical properties suitable for biomedical applications.

## 7. Conclusions

Using the chitosan and silk fibroin mixture, one can fabricate membranes, wound dressings, artificial skin, and implantable devices for the delivery of biologically active compounds. The blends of chitosan and silk fibroin containing inorganic particles and/or nanoparticles have potential for applications as implants of soft and hard tissues. Although many research groups work with chitosan and silk fibroin blends, there is still a need for new research because the market expects new materials for biomedical applications. Undeniably, chitosan and silk fibroin are very interesting biopolymers for blend preparations. The expectations from the biomedical and cosmetic industries are huge, so probably in the near future materials based on chitosan and silk fibroin blends will also be available on the market.

## Data Availability

Not applicable.
